# Measure of Significance of Holotropic Breathwork in the Development of Self-Awareness

**DOI:** 10.1089/acm.2014.0297

**Published:** 2015-12-01

**Authors:** Tanja Miller, Laila Nielsen

**Affiliations:** ^1^ Aalborg University, Aalborg, Denmark.; ^2^ Psykolog Praksis, Snedsted, Denmark.

## Abstract

***Objectives:*** To investigate whether Holotropic Breathwork™ (HB; Grof Transpersonal Training, Mill Valley, CA) has any significance in the development of self-awareness.

***Design:*** A quasi-experiment design and multiple case studies. A single case design was replicated. The statistical design was a related within-subject and repeated-measures design (pre-during-post design).

***Setting/location:*** The study was conducted in Denmark.

***Participants:*** The participants (*n =* 20) were referred from Danish HB facilitators. Nine were novices and 11 had experience with HB.

***Intervention:*** Four HB sessions.

***Outcome measures:*** The novices (*n* = 9) underwent positive temperament changes and the experienced participants (*n* = 11) underwent positive changes in character. Overall, positive self-awareness changes were indicated; the participants' (*n* = 20) scores for persistence temperament, interpersonal problems, overly accommodating, intrusive/needy, and hostility were reduced. Changes in temperament were followed by changes in paranoid ideation scale, indicating a wary phase.

***Results:*** Participants (*n* = 20) experienced reductions in their persistence temperament scores. The pretest mean (mean ± standard deviation, 114.15 ± 16.884) decreased at post-test (110.40 ± 16.481; pre–during-test *p =* 0.046, pre–post-test *p =* 0.048, pre–post-test effect size [*d*]* =* 0.2). Temperament changes were followed by an increase in paranoid ideation; the pre-test mean (47.45 ± 8.88) at post-test had increased to a higher but normal score (51.55 ± 7.864; pre–during-test *p =* 0.0215, pre–post-test *p =* 0.021, pre–post-test *d =* 0.5). Pre-test hostility mean (50.50 ± 10.395) decreased at post-test (47.20 ± 9.001; *p =* 0.0185; *d =* 0.3). The Inventory of Interpersonal Problems total pre-test mean (59.05 ± 17.139) was decreased at post-test (54.8 ± 12.408; *p =* 0.044; *d =* 0.2). Overly accommodating pre-test mean (56.00 ± 12.303) was decreased at post-test (51.55 ± 7.797; *p =* 0.0085; *d =* 0.4). The intrusive/needy pre-test score (57.25 ± 13.329) was decreased at post-test (52.85 ± 10.429; *p =* 0.005; *d =* 0.4).

***Conclusions:*** The theoretical conclusion is that HB can induce very beneficial temperament changes, which can have positive effects on development of character, measured as an increase in self-awareness.

## Introduction

Christina and Stanislav Grof developed Holotropic Breathwork™ (HB; Grof Transpersonal Training, Mill Valley, CA) in 1975. HB is a psychotherapeutic procedure involving hyperventilation, a voluntary, prolonged, mindful, and deep overbreathing procedure supported by music and elective bodywork. The HB session is largely nonverbal and without interventions. It concludes with mandala drawing and sharing. A typical HB session lasts for about 1–3 hours, and the client terminates the session voluntarily.^1,2^

The research on HB is sparse. Rhinewine and Williams^3^ found only three studies that appear to constitute reliable and empirical evidence. Holmes and colleagues' research was published in a peer-reviewed journal.^4^ Pressman's PhD thesis (1993) and Hanratty's PhD thesis (2002) are unpublished.^3^

The primary purpose of this pilot study was to examine empirically the therapeutic value of HB. The research question was, Does HB have any significance in the development of self-awareness, and, in that case, what kind of significance does it have?

## Materials and Methods

### Ethical approval

The Regional Committee on Health Research Ethics for Northern Jutland, Videnskabsetiske Komite for Region Nordjylland, concluded that the project was a questionnaire and interview study rather than an interventional study and was not part of a study of biological material. Therefore, notifying the Committee on Health Research Ethics was not required. The Danish Data Protection Agency (Datatilsynet) approved the project on October 5, 2009 (reference no. 2009-41-3807). The participants gave informed consent.

### Participants

All participants were referred by HB facilitators, who offered free HB sessions for this study. The facilitators advertised the research project on their website homepages, and potential participants registered their interest in the project via these homepages. Twenty participants participated (All-HB). Exclusion criteria were previous HB sessions with the researcher or contraindications for HB as specified by the HB facilitators: glaucoma, retinal detachment, osteoporosis, cardiovascular disease (including heart attacks, angina, and high blood pressure), aneurysm, communicable or infectious diseases, seizure disorders, strong medication, severe mental illness, recent significant surgery or injuries, and pregnancy.^1^

Participants included 11 women and 9 men. Nine participants were novices (no HB experience; 0-HB group). Eleven participants had previously undergone 1–40 HB sessions, for a mean of 6.5 sessions (Exp-HB group). The participants' ages ranged from 25 to 56 years (mean age, 44.25 years). The participants' educational background was as follows: Twenty-five percent had attended vocational training or high school, 5% had undergone a short period of higher education (<3 years), 40% had 3–4 years of education, and 30% had more than 4 years of education (e.g., a Master's or PhD degree). Seven participants dropped out: Two did not complete the questionnaires on time, and five withdrew because of illness and logistic problems.

### Study design

A quasi-experiment design was chosen because random assignment of participants was not possible in this field study. To make the analytical conclusions more powerful,^5^ the chosen method was a multiple-case study in which a single-case design was replicated. The statistical design^6^ was a related within-subject and repeated-measures design (pre-during-post design). Eighteen persons subsequently participated in a semi-structured interview.

### Intervention

The participants (*n* = 20) engaged in four HB sessions, which took place during two weekend workshops separated by a 12-week interval. They underwent two HB sessions at each weekend workshop.

### Measures

The Temperament and Character Inventory (TCI-R), validated by Cloninger, was used to find indications of significant movement in self-awareness. Implicit in Cloninger's works are measurements of self-awareness levels regarding subject-subject relations, subject-object relations, and object-object relations. TCI-R measures four types of temperaments (novelty seeking, harm avoidance, reward dependence, and persistence) and three character scales (self-directedness, cooperativeness, and self-transcendence). According to Cloninger, it is most advantageous to have average temperament scores because they are often connected to an organized character. Temperament refers to the emotional response we have automatically. High character scores indicate high self-awareness, maturity, and a well-regulated personality, which is also connected to well-being.^7^ The Danish TCI-R version and raw score were used.^*,8^

Several sources were used to raise the construct validity. To discover possible changes in the subject-object relation and identify the participants' interpersonal problems, the validated Inventory of Interpersonal Problems (IIP) was applied. The following scales describe these interpersonal difficulties: domineering/controlling, vindictive/self-centered, cold/distant, socially inhibited, nonassertive, overly accommodating, self-sacrificing, and intrusive/needy. The IIP total T score in general indicates levels of interpersonal mental distress.^9^

To identify changes in the object-object relation, the validated Symptom Checklist (SCL-90-R) was applied, measuring the extent of symptoms. SCL-90-R has the following scales: somatization, obsessive-compulsive, interpersonal sensitivity, depression, anxiety, hostility, phobic anxiety, paranoid ideation, and psychoticism. The Global Severity Index measures the overall psychological distress. The Positive Symptom Distress Index measures the intensity of symptoms and a Positive Symptom Total score and records the number of self-reported symptoms.^10^

**FIG. 1.**
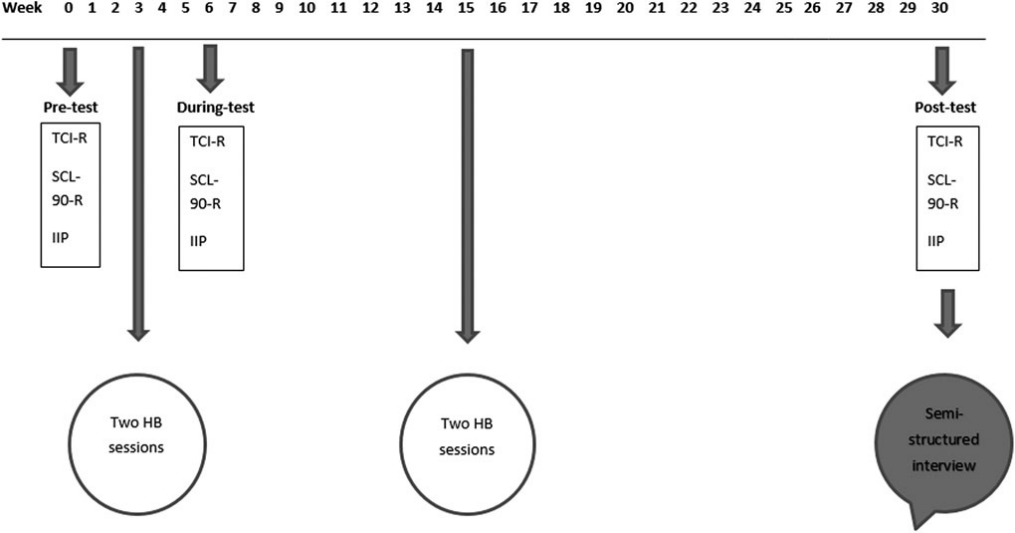
Time flow of the completion of the questionnaires, the Holotropic Breathwork (HB) sessions at weekend workshops, and the semi-structured interview. Questionnaires were the Temperament and Character Inventory R (TCI-R), Symptom Checklist-90-R (SCL-90-R), and Inventory of Interpersonal Problems (IIP).

To follow the continuously nonlinear unfolding of the self-awareness phenomenon, repeated measures were used. The TCI-R, IIP, and SCL-90-R questionnaires were completed 3 weeks before the first two HB sessions (pre-test), 3 weeks after these first two HB sessions (during-test), and 15 weeks after the fourth HB session (post-test) (Fig. 1).

### Statistical analysis

Analyses were performed using SPSS versions 20 and 22. The repeated measure data from the TCI-R, IIP, SCL-90-R questionnaires were statistically analyzed using the nonparametric Wilcoxon *T* test for related samples. The test is a distribution-free test^6^ used at an ordinal level.

**FIG. 2.**
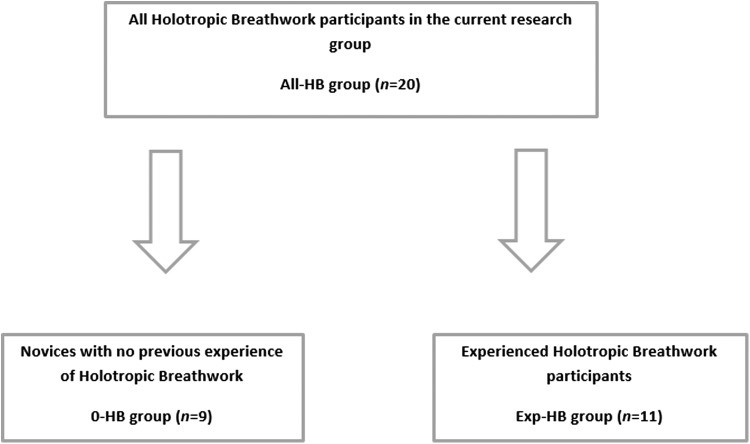
Distribution of HB novices and more experienced HB participants.

The effect size was measured using Cohen *d,*^11^ in which SD is calculated as a pooled variance estimate. Descriptive statistics were provided for age, education, and experience with HB. Results were provided for the All-HB group (*n* = 20), the 0-HB group (*n* = 9), and the Exp-HB group (*n* = 11) (Fig. 2).

## Results

For the All-HB group (*n* = 20), positive changes in mean pre-post were found on 24 of 28 scales. Four scales moved within the average score range in a less favorable direction (Table 1).

**Table 1.** Results for All–Holotropic Breathwork Group (*n* = 20): Pre-During and Pre-Post Significance, Effect Size, Mean ± Standard Deviation

	p*-Value*					
*Measures*	*Pre-during*^a^	*Pre-post*^a^	*Effect size (*d*)*^b^*pre-post*	*Pre-test mean ± SD*	*During-test mean ± SD*	*Post-test mean ± SD*
Novelty seeking^c^	0.1685	0.2855		112.5 ± 10.797	112.95 ± 8.793	111.65 ± 7.896
Harm avoidance^c^	0.4045	0.199		97.00 ± 16.556	97.05 ± 16.12	95.20 ± 17.57
Reward dependence^c^	0.307	0.3435		106.00 ± 11.475	106.75 ± 11.648	105.00 ± 9.032
Persistence^c^	0.046^d^	0.048^d^	0.2 small	114.15 ± 16.884	111.05 ± 18.497	110.40 ± 16.481
Self-directedness^c^	0.389	0.3145		139.35 ± 22.210	139.65 ± 21.772	139.60 ± 19.011
Cooperativeness^c^	0.036^d^	0.5		143.40 ± 13.690	141.05 ± 13.96	143.30 ± 12.511
Self-transcendence^c^	0.1875	0.07		84.15 ± 14.922	82.15 ± 16.246	86.55 ± 15.919
IIP total^e^	0.1905	0.044^d^	0.2 small	59.05 ± 17.139	57.65 ± 15.332	54.80 ± 12.408
Domineering/controlling^e^	0.4875	0.086		55.15 ± 16.872	55.60 ± 14.292	51.40 ± 12.258
Vindictive/self-centered^e^	0.2365	0.074		55.40 ± 13.751	54.35 ± 12.596	51.50 ± 9.157
Cold/distant^e^	0.412	0.0845		60.55 ± 17.911	59.80 ± 16.52	56.50 ± 14.036
Socially inhibited^e^	0.071	0.3865		56.20 ± 12.099	54.65 ± 12.704	55.65 ± 11.245
Nonassertive^e^	0.285	0.2455		57.50 ± 13.539	56.65 ± 11.618	55.95 ± 9.622
Overly accommodating^e^	0.216	0.0085^d^	0.4 small	56.00 ± 12.303	54.90 ± 12.540	51.55 ± 7.797
Self-sacrificing^e^	0.1725	0.229		52.50 ± 12.718	51.55 ± 10.195	50.50 ± 11.255
Intrusive/needy^e^	0.1475	0.005^d^	0.4 small	57.25 ± 13.329	55.25 ± 13.094	52.85 ± 10.429
Global Severity Index^f^	0.4925	0.336		55.75 ± 8.422	55.65 ± 9.461	55.05 ± 8.432
Positive Symptom Distress Index^f^	0.1045	0.2165		55.75 ± 9.781	53.50 ± 9.902	53.60 ± 9.344
Positive Symptom Total^f^	0.2915	0.4775		55.85 ± 8.248	56.40 ± 8.419	55.70 ± 7.881
Somatization^f^	0.3585	0.468		53.40 ± 11.381	52.20 ± 11.786	53.50 ± 8.495
Obsessive-compulsive^f^	0.343	0.1475		54.75 ± 8.397	53.85 ± 10.363	52.85 ± 10.806
Interpersonal sensitivity^f^	0.3215	0.099		55.90 ± 9.130	56.55 ± 9.133	54.25 ± 9.066
Depression^f^	0.279	0.2995		57.05 ± 8.407	55.55 ± 10.211	55.85 ± 9.241
Anxiety^f^	0.4475	0.4795		55.05 ± 8.876	54.00 ± 9.386	54.85 ± 8.054
Hostility^f^	0.3245	0.0185^d^	0.3 small	50.50 ± 10.395	49.85 ± 11.338	47.20 ± 9.001
Phobic anxiety^f^	0.2855	0.323		54.55 ± 11.114	55.05 ± 11.927	53.80 ± 11.228
Paranoid ideation^f^	0.0215^c^	0.021^d^	0.5 medium	47.45 ± 8.882	50.25 ± 10.857	51.55 ± 7.864
Psychoticism^f^	0.35	0.211		54.80 ± 11.919	55.85 ± 11.568	56.20 ± 9.203

^a^ One-tailed.

^b^ Effect size *d* = (*M1* − *M2*)/SD. SD is calculated as a pooled variance estimate: $SD = \sqrt { ( ( ( n1 - 1 ) \times SD1^2 + ( n2 - 1 ) \times SD2^2 ) / ( n1 + n2 ) ) }$, where *n1* is pre-number, *n2* is post-number, *SD1* is SD pre, *SD2* is SD after)).

^c^ Temperament and Character Inventory. American psychometric and normative data were provided by Robert Cloninger, MD, Thomas R. Przybeck, PhD, Dragan M. Svrakic, MD, PhD, Richard D. Wetzel, PhD (1994). The TCI scale's Cronbach was moderately to highly reliable.^12^ Danish TCI-R translation was provided by Ann Suhl Kristensen, PhD, and Ole Mors, PhD.^*,8^ Raw score was used because validity indicators were not yet available in Danish.

^d^ The difference was significant at ≤ 5%.

^e^ Inventory of Interpersonal Problems (IIP) by Leonard M. Horowitz, PhD (2008). Danish edition was used, in which the IIP scale's Cronbach'sα was moderately to highly reliable.^9^

^f^ Symptom Checklist-90-R (SCL-90-R) by Leonard R. Derogatis, PhD (2009). Danish edition was used, in which the SCL-90-R scale's Cronbach'sα was moderately to highly reliable.^10^

SD, standard deviation.

### TCI-R: significant changes for the All-HB group (n = 20)

The mean persistence temperament for the All-HB group at pre-test was close to the high score (mean ± standard deviation, 114.15 ± 16.884), and it changed at post-test toward a more beneficial score (110.40 ± 16.481). There were significant temperament changes at pre–during-test (*p =* 0.046) and significant changes at pre–post-test (*p =* 0.048), where the effect size was small (*d =* 0.2). Cooperativeness decreased from pre-test (143.40 ± 13.690) to during-test (141.05 ± 13.960), a significant difference (*p =* 0.036). At pre–during-test, but not pre–post-test, the mean returned to baseline (143.30 ± 12.511). Cooperativeness pre-during effect size was (*d* = 0.17).

### SCL-90-R: significant changes for the all-HB group (n = 20)

For the All-HB group, the temperament changes were followed by an increase in paranoid ideation. The pre-test mean was below a T score of 50 (47.45 ± 8.882), but at post-test it had increased to a higher yet still normal score (51.55 ± 7.864). The change was significant at both pre–during-test (*p =* 0.0215) and pre–post-test (*p =* 0.021), and the effect size at pre-post was medium (*d =* 0.5).

For the All-HB group, hostility was average at baseline but decreased further. Mean pre-test hostility (50.50 ± 10.395) was decreased at post-test (47.20 ± 9.001). This reduction was significant (*p =* 0.0185), and the effect size was small (*d =* 0.3).

### IIP: significant changes for the All-HB group (n = 20)

The total IIP pre-test mean for the All-HB group was close to the high score (59.05 ± 17.139) and at post-test had become more favorable (54.8 ± 12.408). Interpersonal problems decreased significantly (total IIP, *p =* 0.044), with a small effect size (*d =* 0.2).

The process for the All-HB group showed that the pre-test mean for overly accommodating (56.00 ± 12.303) was decreased at post-test (51.55 ± 7.797). This reduction was significant (*p =* 0.0085), and the effect size was small (*d =* 0.4).

The intrusive/needy score at pre-test was close to a high score (57.25 ± 13.329) but was decreased at post-test close to average (52.85 ± 10.429). The reduction for the All-HB group was significant (*p =* 0.005), with a small effect size (*d =* 0.4).

### Investigation of significant changes for the 0-HB group (n = 9)

When the scores for the 0-HB group were extracted, the self-awareness process for these novices developed in a different direction compared with that of the experienced HB group. The 0-HB group had high pre-test temperament scores for novelty seeking (118.78 ± 8.497), reward dependence (108.00 ± 11.673), and persistence (124.00 ± 13.435). At post-test, the temperament scores were average for novelty seeking (113.56 ± 6.894) and persistence (115.11 ± 10.055). The reward dependence scores decreased toward the average but were still high (105.67 ± 7.211).

There were significant temperament changes at pre-post for novelty seeking (*p =* 0.0245), and the effect size was medium (*d =* 0.7). There was also a significant reduction in persistence (*p =* 0.0255), with a large effect size (*d =* 0.8).

**FIG. 3.**
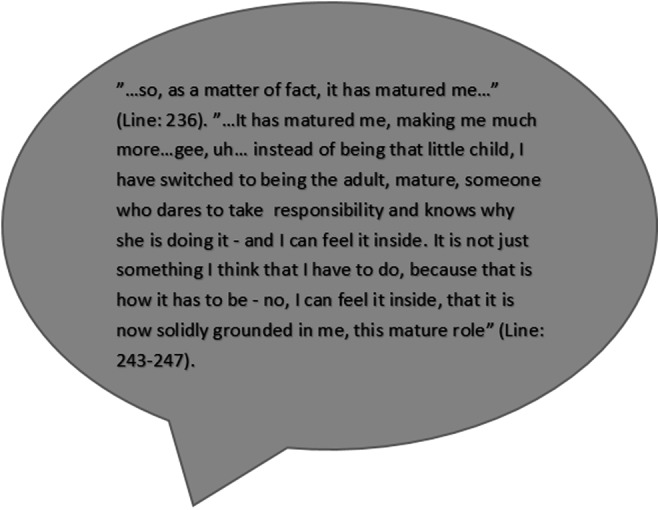
A quote from a semi-structured interview with Karen, age 54 years, no HB experience.

Harm avoidance was average at pre-test (97.11 ± 14.802) and was decreased at during-test (92.89 ± 15.640), a significant change (pre–during-test, *p =* 0.022), and the effect size was small (*d =* 0.3). At post-test, the mean was still within the average range and decreased even further (91.22 ± 18.559), but it was only close to significant (Fig. 3).

In general, the results for the 0-HB group indicated a new temperament baseline, which could be a more advantageous prerequisite for organized character development. The temperament changes for the 0-HB group were followed by a reduction in socially inhibited problems, wherein the pre-test T score (55.22 ± 10.269) was reduced at during-test (52.22 ± 12.397). This change was significant (pre–during-test *p =* 0.0455), and the effect size was small (*d =* 0.3). The pre–post-test difference was not significant, although the problems were reduced at post-test (53.67 ± 9.513).

The temperament change for 0-HB group is combined with an increase in paranoid ideation. At pre-test, the 0-HB group was within the normal range of paranoid ideation symptoms (45.89 ± 7.785); at post-test the symptoms were increased but were still within the normal range (52.78 ± 9.025). This change was significant (*p =* 0.025) and the effect size was large (*d =* 0.9). This finding indicates that for the 0-HB group, when medium and large changes in temperament were seen, participants were wary in this initial phase. When significant changes take place for the novices and resulted in small changes in harm avoidance at pre–during-test, the participants simultaneously reported that they had become less socially inhibited (Table 2).

**Table 2.** Results for 0-HB Group (*n* = 9) (No Previous Holotropic Breathwork Experience): Pre-During and Pre-Post Significance, Effect Size, Mean ± Standard Deviation

	p*-Value*					
*Measures*	*Pre-during*^a^	*Pre-post*^a^	*Effect size (*d*)*^b^	*Pre-test mean ± SD*	*During-test mean ± SD*	*Post-test mean ± SD*
Novelty seeking^8^	0.124	0.0245^c^	0.7 medium	118.78 ± 8.497	116.22 ± 7.362	113.56 ± 6.894
Harm avoidance^8^	0.022^c^	0.0705	0.3 small	97.11 ± 14.802	92.89. ± 15.640	91.22. ± 18.559
Reward dependence^8^	0.363	0.1865		108.00 ± 11.673	107.00 ± 10.380	105.67 ± 7.211
Persistence^8^	0.275	0.0255^c^	0.8 large	124.00 ± 13.435	121.11 ± 14.650	115.11 ± 10.055
Self-directedness^8^	0.297	0.1175		138.00 ± 21.994	140.11 ± 21.368	139.78 ± 14.771
Cooperativeness^8^	0.086	0.096		145.56 ± 7.485	142.44 ± 9.964	140.56 ± 9.180
Self-transcendence^8^	0.3895	0.476		86.33 ± 13.946	85.33 ± 14.874	86.11 ± 15.862
IIP total^9^	0.075	0.1165		58.22 ± 16.415	56.11 ± 14.954	54.00 ± 9.975
Domineering/controlling^9^	0.367	0.457		55.00 ± 16.636	56.67 ± 16.163	53.33 ± 12.913
Vindictive/self-centered^9^	0.1985	0.305		53.00 ± 10.50	51.00 ± 9.836	50.67 ± 5.568
Cold/distant^9^	0.429	0.22		54.89 ± 12.937	54.56 ± 11.469	51.44 ± 8.516
Socially inhibited^9^	0.0455^c^	0.3335	0.3 small	55.22 ± 10.269	52.22 ± 12.397	53.67 ± 9.513
Nonassertive^9^	0.088	0.13		58.33 ± 13.105	55.33 ± 12.600	54.33 ± 9.772
Overly accommodating^9^	0.146	0.2		56.33 ± 12.961	53.67 ± 12.981	53.11 ± 8.298
Self-sacrificing^9^	0.416	0.433		53.22 ± 14.898	53.11 ± 11.548	51.67 ± 11.554
Intrusive/needy^9^	0.416	0.103		58.44 ± 16.118	57.22 ± 15.699	53.78 ± 11.617
Global Severity Index^10^	0.296	0.5		54.22 ± 8.228	53.11 ± 7.339	54.33 ± 8.803
Positive Symptom Distress Index^10^	0.0705	0.312		54.78 ± 9.718	49.22 ± 7.412	51.89 ± 7.288
Positive Symptom Total^10^	0.4155	0.1865		54.67 ± 8.352	54.89 ± 6.679	55.89 ± 9.103
Somatization^10^	0.117	0.2875		54.78 ± 10.721	50.00 ± 9.083	55.00 ± 8.660
Obsessive-compulsive^10^	0.2555	0.363		53.22 ± 7.530	50.56 ± 9.567	52.00 ± 13.086
Interpersonal sensitivity^10^	0.187	0.2415		53.56 ± 8.676	55.00 ± 5.916	54.67 ± 7.566
Depression^10^	0.264	0.3365		54.67 ± 8.047	52.78 ± 6.610	52.89 ± 9.307
Anxiety^10^	0.472	0.4165		54.56 ± 8.110	52.33 ± 10.173	54.33 ± 8.617
Hostility^10^	0.3675	0.2995		49.33 ± 8.170	48.67 ± 10.198	48.33 ± 6.745
Phobic anxiety^10^	0.5	0.1365		52.11 ± 10.588	52.44 ± 10.956	54.44 ± 10.795
Paranoid ideation^10^	0.1345	0.025^c^	0.9 large	45.89 ± 7.785	48.33 ± 8.930	52.78 ± 9.025
Psychoticism^10^	0.4325	0.2415		54.56 ± 10.248	52.89 ± 8.937	56.00 ± 8.201

^a^ One-tailed.

^b^ Effect size *d* = (*M1* − *M2*)/SD. SD is calculated as a pooled variance estimate: $SD = \sqrt { ( ( ( n1 - 1 ) \times SD1^2 + ( n2 - 1 ) \times SD2^2 ) / ( n1 + n2 ) ) }$, where *n1* is pre-number, *n2* is post-number, SD1 is SD pre, SD2 is SD after)).

^c^ The difference was significant at ≥ 5%.

### Investigation of significant changes for the Exp-HB group (n = 11)

Temperament change was seen at pre–during-test because the novelty seeking mean for Exp-HB group at pre-test (107.36 ± 9.963) increased at during-test (110.27 ± 9.275), a score within the average range. There was a significant change in temperament at pre–during-test (*p =* 0.026), and the effect size was small (*d =* 0.3), but at post-test (110.09 ± 8.631), the change was not significant.

The persistence mean for the Exp-HB group decreased from pre-test (106.09 ± 15.443) to during-test (102.82 ± 17.685). This temperament change was significant at pre–during-test (*p =* 0.0495), and the effect size was small (*d* = 0.2). There was no significant change at pre–post-test. The mean returned to pre-test level at post-test (106.55 ± 19.972).

When temperament changes occurred for the Exp-HB group, there was a simultaneous increase in the mean paranoid ideation at pre–during-test. The pre-test mean (48.73 ± 9.870) was increased at during-test but was still within the normal range (51.82 ± 12.416); this was not a significant change but was close to significant.

The novelty seeking temperament changes for the Exp-HB group were not significant at pre–post-test, and the mean paranoid ideation decreased simultaneously at post-test (50.55 ± 7.062); however, there was no significant change at pre–post-test.

The results for the 0-HB group and the Exp-HB group indicated that as long as small, medium, and large temperament changes took place at pre–post-test, the novices experienced a new automatic emotional response. This seemed to make them more wary. The results indicated that with more HB experience the temperament change settled and the wary phase waned because there was no significant change in temperament at pre–post-test and no significant changes in paranoid ideation at pre–post-test for the Exp-HB group.

### Further investigation of significant changes for the Exp-HB group (n = 11)

The Exp-HB group had no significant temperament changes at pre–post-test. Instead, the participants underwent positive character changes; the mean self transcendence score increased from pre-test (82.36 ± 16.114) to post-test (86.91 ± 16.730), and this change was significant at pre–post-test (*p =* 0.0225), with a small effect size (*d =* 0.3).

**FIG. 4.**
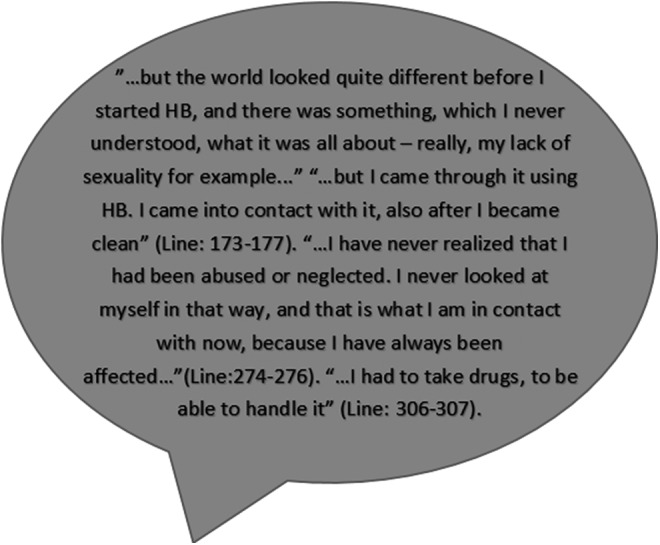
A quote from the semi-structured interview with Jane, age 56 years, addicted to drugs for 40 years and clean for the past 2.5. She had completed 10 HB sessions.

The cooperativeness mean at pre-test (141.64 ± 17.426) for the Exp-HB group also increased at post-test (145.55 ± 14.754), but this change was only close to significant (*p =* 0.0625) (Fig. 4).

These results indicate higher self-awareness in both object-object relations and subject-object relations and are supported by a reduction in the Exp-HB groups domineering/controlling problems from pre-test (55.27 ± 17.872) to post-test (49.82 ± 12.082), at which point the problems were absent. This change in domineering/controlling was significant at pre–post-test (*p =* 0.045), and the effect size was small (*d =* 0.4).

The positive change in self-awareness was also supported by the reduction in the Exp-HB group's, overly accommodating problems. The mean at pre-test (55.73 ± 12.370) was reduced at post-test (50.27 ± 7.511), which was significant at pre–post-test (*p =* 0.0135), with a medium effect size (*d =* 0.6).

In addition, intrusive/needy problems decreased for the Exp-HB group, as can be seen from pre-test (56.27 ± 11.288) to post-test (52.09 ± 9.864). This reduction in intrusive/needy problems was a positive, significant change in the Exp-HB group at pre–post-test (*p =* 0.0055), and the effect size was small (*d =* 0.4).

In addition, the Exp-HB group had experienced a reduction in interpersonal sensitivity symptoms from pre-test (57.82 ± 9.443) to a more advantageous mean at post-test (53.91 ± 10.492). The change was significant at pre-post (*p =* 0.023), with a small effect size (*d =* 0.4).

Further symptom reduction supported a higher self-awareness for the Exp-HB group. The hostility pre-test mean (51.45 ± 12.234) also became a positive mean at post-test (46.27 ± 10.743), and this was a significant change at pre–post-test (*p =* 0.0155), with a medium effect size (*d =* 0.5) (Table 3).

**Table 3.** Results for the Experienced Holotropic Breathwork Group (*n* = 11): Pre-During and Pre-Post Significance, Effect Size, Mean ± Standard Deviation

	p*-Value*					
*Measures (Reference)*	*Pre-during*^a^	*Pre-post*^a^	*Effect size (*d*)*^b^	*Pre-test mean ± SD*	*During mean ± SD*	*Post-test mean ± SD*
Novelty seeking^8^	0.026^c^	0.11	0.3 small	107.36 ± 9.963	110.27 ± 9.275	110.09 ± 8.631
Harm avoidance^8^	0.062	0.3445		96.91 ± 18.587	100.45 ± 16.422	98.45 ± 16.884
Reward dependence^8^	0.152	0.4795		104.36 ± 11.604	106.55 ± 13,095	104.45 ± 10.615
Persistence ^8^	0.0495^c^	0.4295	0.2 small	106.09 ± 15.443	102.82 ± 17.685	106.55 ± 19.972
Self-directedness^8^	0.439	0.4795		140.45 ± 23.394	139.27 ± 23.130	139.45 ± 22.629
Cooperativeness^8^	0.0915	0.0625		141.64 ± 17.426	139.91 ± 16.961	145.55 ± 14.754
Self-transcendence^8^	0.1635	0.0225^c^	0.3 small	82.36 ± 16.114	79.55 ± 17.546	86.91 ± 16.730
IIP total^9^	0.453	0.0765		59.73 ± 18.478	58.91 ± 16.245	55.45 ± 14.556
Domineering/controlling^9^	0.3675	0.045^c^	0.4 small	55.27 ± 17.872	54.73 ± 13.312	49.82 ± 12.082
Vindictive/self-centered^9^	0.428	0.0765		57.36 ± 16.176	57.09 ± 14.342	52.18 ± 11.548
Cold/distant^9^	0.36	0.1405		65.18 ± 20.571	64.09 ± 19.191	60.64 ± 16.567
Socially inhibited^9^	0.3215	0.4795		57.00 ± 13.864	56.64 ± 13.193	57.27 ± 12.705
Nonassertive^9^	0.312	0.383		56.82 ± 14.483	57.73 ± 11.252	57.27 ± 9.758
Overly accommodating^9^	0.4795	0.0135^c^	0.6 medium	55.73 ± 12.370	55.91 ± 12.708	50.27 ± 7.511
Self-sacrificing^9^	0.155	0.106		51.91 ± 11.353	50.27 ± 9.318	49.55 ± 11.475
Intrusive/needy^9^	0.2065	0.0055^c^	0.4 small	56.27 ± 11.288	53.64 ± 11.057	52.09 ± 9.864
Global Severity Index^10^	0.2805	0.224		57.00 ± 8.764	57.73 ± 10.790	55.64 ± 8.500
Positive Symptom Distress Index^10^	0.406	0.2705		56.55 ± 10.231	57.00 ± 10.602	55.00 ± 10.890
Positive Symptom Total^10^	0.2865	0.224		56.82 ± 8.436	57.64 ± 9.760	55.55 ± 7.188
Somatization^10^	0.193	0.2115		52.27 ± 12.289	54.00 ± 13.784	52.27 ± 8.568
Obsessive-compulsive^10^	0.4595	0.1525		56.00 ± 9.209	56.55 ± 10.634	53.55 ± 9.147
Interpersonal sensitivity^10^	0.3225	0.023^c^	0.4 small	57.82 ± 9.443	57.82 ± 11.250	53.91 ± 10.492
Depression^10^	0.4645	0.3605		59.00 ± 8.556	57.82 ± 12.270	58.27 ± 8.867
Anxiety^10^	0.4795	0.305		55.45 ± 9.832	55.36 ± 8.947	55.27 ± 7.964
Hostility^10^	0.3115	0.0155^c^	0.5 medium	51.45 ± 12.234	50.82 ± 12.600	46.27 ± 10.743
Phobic anxiety^10^	0.246	0.084		56.55 ± 11.631	57.18 ± 12.774	53.27 ± 12.067
Paranoid ideation^10^	0.051	0.1865		48.73 ± 9.870	51.82 ± 12.416	50.55 ± 7.062
Psychoticism^10^	0.1725	0.337		55.00 ± 13.631	58.27 ± 13.267	56.36 ± 10.347

^a^ One-tailed.

^b^ Effect size *d* = (*M1* − *M2*)/SD. SD is calculated as a pooled variance estimate: $SD = \sqrt { ( ( ( n1 - 1 ) \times SD1^2 + ( n2 - 1 ) \times SD2^2 ) / ( n1 + n2 ) ) }$, where *n1* is pre-number, *n2* is post-number, *SD1* is SD pre, *SD2* is SD after.

^c^ The difference was significant at minimum 5%.

## Discussion

For the All-HB group (*n =* 20), significant temperament changes were seen for persistence. This indicates a movement toward a temperament that, according to Cloninger,^7^ is connected to a lower risk of obsessional tendencies, which can make it easier to handle contingency events. The All-HB group experienced a significant reduction in interpersonal problems and IIP total score, which indicates that participants became more sociable and experienced less interpersonal mental distress in general.

The study results show that the self-awareness process for the nine novices primarily led to significant positive medium and large changes in temperament at pre-post.

The results for the more HB-experienced group primarily show character changes because there were positive significant changes at pre–post-test on the character scale self-transcendence. The positive self-awareness changes were supported by a significant reduction in interpersonal problems, such as domineering/controlling, overly accommodating, intrusive needy, interpersonal sensitivity, and hostility symptoms.

This finding suggests that novices with high temperament scores may be prepared to undergo major positive changes in their automatic emotional responses when they start practicing HB. The results indicate that the self-awareness process, with more HB experience, continues in a positive direction toward a significant positive character development and higher self-awareness, as measured with the self-transcendence scale. According to Cloninger,^7^ an increase in self-transcendence indicates that people become more sensible, idealistic, transpersonal, faithful, flexible, self-forgetful, and creative with a higher spiritual awareness. The increase in self-transcendence indicates that participants have become more wise and patient and that they experience more equanimity. It also reflects that participants find it easier to let go of conflicts and struggles about control and being controlled.

HB can induce profound positive temperament changes, which may call for a redefinition of character and the way one directs one's intentions. In this phase, where temperament changes take place, it may cause participants to become more wary because their automatic reactions are new. For the novices, this indicates profound beneficial changes in temperament as they move from a high score on novelty seeking and persistence to an average score, which can decrease the risk of immaturity. An average temperament score is, according to Cloninger,^7^ connected to a lower risk of immaturity. Cloninger describes temperament as developmentally stable with increasing age, pharmacotherapy, and psychotherapy,^7^ which makes these results remarkable.

The study is based on typical conditions for HB practice. However, the study has several limitations. The sample size was small; with regard to the multiple comparisons of the 0-HB and Exp-HB groups, this is especially a limitation. The participants were volunteers who already had an interest in HB. The volunteers might have had significantly different characteristics than the norm.^6^ Moreover, the study's participants were not randomly selected, and the study results cannot be generalized to a larger population because there is a bias compared to the composition of the general population. The external validity is low because the sample is not representative; thus, according to Yin,^5^ it is only appropriate to use the findings to make analytical generalizations. Several validated questionnaires sources were used to raise the construct validity, and they were handled reliably using case study protocol and databases. With regard to the statistical procedure for the present sample, the internal validity is high.

The theoretical conclusion is that HB can induce significantly large beneficial temperament changes, which was the case for the group of novices with high persistence temperament scores. For the novice group, HB significantly reduced high temperament score on novelty seeking, which is connected to a lower risk of immaturity. The biggest temperament changes can simultaneously be followed by a significantly more wary phase because the participant's automatic response is new.

For the group with more HB experience, a significant increase in self-transcendence score was found, which indicates a higher self-awareness. For the experienced group, this is supported by a significant reduction in overly accommodating, intrusive/needy, domineering/controlling problems, and hostility and interpersonal sensitivity symptoms.

The four HB sessions significantly reduced the whole group's (*n* = 20) scores with regard to persistence, hostility, and interpersonal problems, including overly accommodating problems and intrusive/needy problems. The temperament change was followed by a wary phase. HB practice can provide a more organized character development measured as progression in the development of self-awareness.

## Notes

^*^ Kristensen AS and Mors O. TCI-R, Center for Psykiatrisk Grundforskning, Aarhus Universitetshospital. September 2004.

^*^ Kristensen AS and Mors O. TCI-R, Center for Psykiatrisk Grundforskning, Aarhus Universitetshospital. September 2004.
